# Hippocampal Estrogen Signaling Mediates Sex Differences in Retroactive Interference

**DOI:** 10.3390/biomedicines10061387

**Published:** 2022-06-11

**Authors:** Marco Rinaudo, Francesca Natale, Francesco La Greca, Matteo Spinelli, Antonella Farsetti, Fabiola Paciello, Salvatore Fusco, Claudio Grassi

**Affiliations:** 1 Department of Neuroscience, Università Cattolica del Sacro Cuore, 00168 Rome, Italy; francesca.natale1@unicatt.it (F.N.); francesco.lagreca@unicatt.it (F.L.G.); matteo.spinelli@unicatt.it (M.S.); fabiola.paciello@unicatt.it (F.P.); salvatore.fusco@unicatt.it (S.F.); claudio.grassi@unicatt.it (C.G.); 2 Fondazione Policlinico Universitario Agostino Gemelli IRCCS, 00168 Rome, Italy; 3 Institute for System Analysis and Computer Science “A. Ruberti” (IASI), National Research Council (CNR), 00185 Rome, Italy; antonella.farsetti@cnr.it

**Keywords:** hippocampus, retroactive interference, estrogens, sex, forgetting, ERK1/2, object recognition memory

## Abstract

Despite being a crucial physiological function of the brain, the mechanisms underlying forgetting are still poorly understood. Estrogens play a critical role in different brain functions, including memory. However, the effects of sex hormones on forgetting vulnerabilitymediated by retroactive interference (RI), a phenomenon in which newly acquired information interferes with the retrieval of already stored information, are still poorly understood. The aim of our study was to characterize the sex differences in interference-mediated forgetting and identify the underlying molecular mechanisms. We found that adult male C57bl/6 mice showed a higher susceptibility to RI-dependent memory loss than females. The preference index (PI) in the NOR paradigm was 52.7 ± 5.9% in males and 62.3 ± 13.0% in females. The resistance to RI in female mice was mediated by estrogen signaling involving estrogen receptor α activation in the dorsal hippocampus. Accordingly, following RI, females showed higher phosphorylation levels (+30%) of extracellular signal-regulated kinase1/2 (ERK1/2) in the hippocampus. Pharmacological inhibition of ERK1/2 made female mice prone to RI. The PI was 70.6 ± 11.0% in vehicle-injected mice and 47.4 ± 10.8% following PD98059 administration. Collectively, our data suggest that hippocampal estrogen α receptor-ERK1/2 signaling is critically involved in a pattern separation mechanism that inhibits object-related RI in female mice.

## 1. Introduction

Memory interference is a widely known phenomenon consisting of either new learning that interferes with previously stored information (retroactive interference, RI) or previous learning interfering with subsequent learning (proactive interference, PrI) [1,2,3]. Numerous studies investigated the mechanisms of memory interference in both animal models and humans, focusing on the key roles of RI and PrI in memory loss (see refs in [4]). Specifically, several works have tried to conceptualize a theoretical framework for interference-mediated forgetting [2] and attempted to dissect the physiological variables and molecular mechanisms underlying forgetting and interference dynamics [5]. At the circuit level, interference involves multiple brain regions that are necessary for memory storage and retrieval [6,7,8]. It has been shown that, in the novel object recognition (NOR) paradigm, exposure to an interference session during the consolidation phase leads to memory loss, and this effect is dependent on medial prefrontal cortex and hippocampal activity [9,10]. Indeed, the hippocampus is thought to store complex representations of events [11,12], while the medial prefrontal cortex biases the retrieval/suppression process [13], selecting the most appropriate memory and, consequently, the most appropriate behavioral response.

Different works have demonstrated that cognitive functions show sexually dimorphic features in both animals and humans. In humans, men tend to outperform women in cognitive tasks in which spatial information plays a crucial role, while females outperform males in verbal-based tasks, showing superior recollection in terms of the speed and accuracy of autobiographical memories [14]. In rodents, behavioral differences have been found in many different cognitive tasks, ranging from the NOR or object place recognition tests to associative paradigms, such as fear conditioning, or spatial navigation paradigms, such as the Morris water maze [15,16,17]. These differences are also substantiated by different long-term potentiation (LTP) induction thresholds, rates of neurogenesis, or different signaling at the synapse, such as the activation of the extracellular signal-regulated kinases 1/2 (ERK1/2), which have been attributed to sex differences in brain development and molecular signaling activated by sex hormones [18,19,20].

The mechanistic regulation of sex-hormone-dependent object memory has been widely described [21], but limited information is available on the gender-based susceptibility to the interference-based object recognition paradigm. Our study aimed to fill this gap. Here, we provide novel evidence that male mice are susceptible to object-related RI memory loss, whereas the memory of female mice is unaffected. The resistance of female mice to RI depends on estrogen α receptor activation and ERK1/2 phosphorylation.

## 2. Materials and Methods

### 2.1. Animals

Wild-type C57BL/6 mice (3–5 months of age) of both sexes, derived from the Animal Facility of Catholic University, were employed for this study. Mice were housed in groups of three to five animals per cage, except after stereotaxic surgeries when they were singly housed. The animals were kept at a controlled temperature of 24 °C under a 12 h light/dark cycle with unrestricted access to food (Mucedola 4RF21, Milan, Italy) and water. A total of 138 C57Bl/6 mice (32 males and 106 females) were used in this study.

### 2.2. Ethics

All animal procedures were approved by the Ethics Committee of Università Cattolica and the Italian Ministry of Health. They were fully compliant with Italian (Legislative Decree No. 26/2014) and European Union (Directive No. 2010/63/UE) legislation on animal research. All efforts were made to limit the number of animals used and to minimize their suffering.

### 2.3. Behavioral Analyses

Behavioral analyses were performed from 9 a.m.to 4 p.m. by an experimenter blind to the treatments. The data were blindly analyzed using the behavioral tracking system ANY-Maze^TM^ (Stoelting Co., Wood Dale, IL, USA). In order to reduce stress-induced manipulations, all animals were handled for 3 min/day during the week preceding the experimental paradigms. Animals undergoing stereotaxic surgeries and intracerebral injections were further exposed to the manual restraint procedure necessary for the injection during the week prior to the test. The objects used for the different object recognition paradigms were: Lego cubes, glass bottles filled with clean bedding, flasks for cell culture filled with clean bedding, and small 3D-printed pyramids. All objects were of similar size.

### 2.4. Standard Novel Object Recognition Paradigm

**Figure 1:**
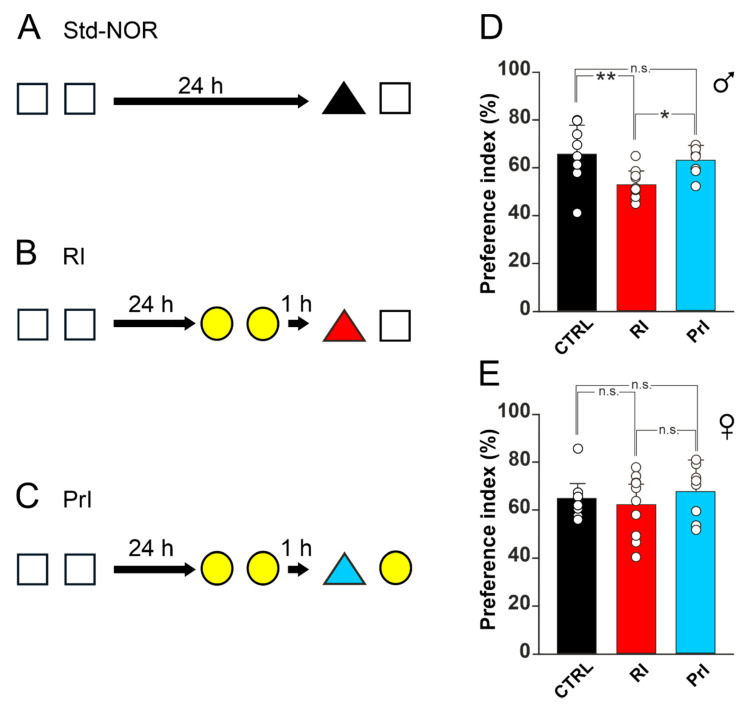
Female mice are resistant to retroactive interference. (**A**–**C**): Schematic representation of different object recognition memory paradigms used. Standard NOR procedure (**A**). Retroactive interference procedure (RI; (**B**)). Proactive interference procedure (PrI; (**C**)); white squares represent object 1, black triangle represents object 2, yellow circles represent object 3, red triangle represents object 4, and blue triangle represents object 5. (**D**): Histograms (mean ± SD) showing preference indexes for male mice undergoing Std-NOR, RI, and PrI. Asterisks indicate statistically significant differences between groups assessed by one-way ANOVA (F_(2;25)_ = 6.731; *p* = 0.005; Std-NOR vs. RI *p* = 0.007, Holm–Sidak post hoc test; Std-NOR vs. PrI *p* = 0.604, Holm–Sidak post hoc test; Std-NOR n = 9; RI n = 11; PrI n = 8). (**E**): Histograms (mean ± SD) showing preference index for female mice undergoing Std-NOR, RI, and PrI. Asterisks indicate statistically significant differences between groups assessed by one-way ANOVA (F_(2;25)_ = 0.590; *p* = 0.562. Std-NOR n = 10; RI n = 10; PrI n = 8). * *p* < 0.05; ** *p* < 0.01; n.s., not significant. All graphs and images were realized using CorelDraw21 (Corel Corporation, Ottawa, Ontario, Canada).

The standard novel object recognition (Std-NOR) paradigm was performed as previously described with slight modifications [22]. The Std-NOR test evaluating object recognition memory consists of a habituation phase, a training phase, and a test phase (Figure 1A). The different phases were separated from each other by 24 h in order to evaluate long-term memory. During the habituation phase, animals were allowed to explore the testing arena (45 × 45 cm) in order to familiarize themselves with it for a total time of 5 min. In the training phase, animals were placed within the testing arena and allowed to explore two identical objects placed symmetrically in the center of the arena for 5 min. During this phase, the exploration time (defined as the time the animal snout was directed at the object from a distance of <2 cm) was recorded to exclude possible preferences for a specific object or side of the arena. Finally, 24 h later, during the test phase, one of the objects was replaced with a novel one, and animals were allowed to explore the objects for 5 more minutes. The exploration time recorded during the test phase was then calculated as the preference index, which is the percentage of time spent exploring the novel object compared to total object exploration (both novel and old objects). To further exclude place preference for one side of the arena, the position of the novel object was alternated on both sides for the different testing sessions. The objects and arena were cleaned with a 70% ethanol solution between the subsequent tests [23].

### 2.5. Interference Paradigm

For the object recognition interference paradigm, which was adapted from [24], animals underwent the same Std-NOR procedure with the addition of an interference session held 24 h after the training phase and 1 h before the testing phase. During the interference session, two identical objects unrelated to the training objects were placed symmetrically in the middle of the arena, and animals were allowed to explore them for 5 min. One hour later, to evaluate the effect of retroactive interference, animals were presented a totally novel object, different from the objects of both training and interference, along with one object from the initial training phase, and they were allowed to explore them for 5 min (Figure 1B). To evaluate proactive interference, in the test phase animals were presented a totally novel object, unrelated to both the training and interference phases, along with one object from the interference phase (Figure 1C). The exploration time was recorded and calculated as previously described for the Std-NOR.

### 2.6. Surgery and Drug Administration

For surgical cannula implantations, animals were deeply anesthetized and placed in a stereotaxic apparatus (Stoelting Co, Wood Dale, IL, USA). To reach the dorsal hippocampus for injections, fixed double guide cannulae (C235G-3.0, Plastics One Inc, Roanoke, VA, USA) were implanted with carboxylate cement (3 M ESPE, Durelon, 3 M Deutschland GmbH, Kamen, Germany) at the following coordinates: posterior to bregma 2.1 mm and lateral to midline ± 1.5 mm. 4-hydroxytamoxifen (4-OHT, H6278, Sigma-Aldrich, Saint Louis, MO, USA), the selective estrogen receptor β antagonist PHTPP (SML1355, Sigma-Aldrich, Saint Louis, MO, USA), the selective estrogen receptor α antagonist MPP (M7068, Sigma-Aldrich, Saint Louis, MO, USA), and the ERK1/2 signaling inhibitor PD98059 (PD-215, Sigma-Aldrich, Saint Louis, MO, USA) were dissolved in dimethyl sulfoxide (DMSO, D5879, Sigma-Aldrich, Saint Louis, USA) and resuspended in water to obtain a final concentration of 10% DMSO. Next, 2 μg of 4-OHT, 1.5 μg of PHTPP and MPP, and 1.5 μg of PD98059 were bilaterally injected into the dorsal hippocampi of female mice. The doses were calculated based on the literature data [25,26,27]. A smaller cannula connected to a polyethylene tube containing the solution to be injected was attached to a 10 μL syringe (Hamilton, NV, USA) that was controlled by an automated pump for infusion. During the injections, animals were manually restrained, and the solution containing the drugs was injected at a flow rate of 0.5 μL/min. Then, 60 or 120 min after the infusion, animals underwent the behavioral procedure. In order to confirm the correct injection site, after the behavioral analysis, the cannula placement was validated with an intrahippocampal injection of methylene blue (1 μL/hippocampus, M9140, Sigma-Aldrich, Saint Louis, MO, USA) followed by visual inspection.

### 2.7. Western Blot

Western blot analyses were performed as previously described [28]. Thirty minutes after interference, animals were sacrificed, and both hippocampi were collected. The tissues were lysed in ice-cold lysis buffer (150 mM NaCl, 50 mM pH 8 Tris-HCl, and 2 mM EDTA) containing 1% Triton X-100, 0.1% sodium dodecyl sulfate, 1 × protease inhibitor cocktail (P8340, Sigma-Aldrich, Saint Louis, MO, USA), 1 mM sodium orthovanadate (S6508, Sigma-Aldrich, Saint Louis, MO, USA), and 1 mM sodium fluoride (201154, Sigma-Aldrich, Saint Louis, MO, USA). After lysis, tissues were spun down at 22,000× *g* and 4 °C, and the supernatants were quantified for protein content (500006, DC protein assay; Bio-Rad, Hercules, CA, USA). Equal amounts of protein were diluted in Laemmli buffer, boiled, and resolved by SDS-PAGE. The primary antibodies were incubated overnight at 4 °C and revealed with HRP-conjugated secondary antibodies (#7074 and #7076, Cell Signaling Technology Inc., Danvers, MA, USA). Primary antibodies for phospho-ERK1/2, total ERK1/2, phospho-Akt, and total Akt (catalogue references, respectively: #9101, #9102, #4060, and #9272, all from Cell Signaling Technology Inc., Danvers, MA, USA) were diluted 1:1000. A primary antibody for tubulin (T6074, Sigma-Aldrich, Saint Louis, MO, USA) was diluted at 1:10,000. Changes in protein phosphorylation were evaluated and documented using UVItec Cambridge Alliance (Uvitec, Cambridge, UK).

### 2.8. Statistical Analyses

Sample sizes were calculated with adequate power (0.8) based on pilot studies and literature data. All statistical analyses were performed by using SigmaPlot 14 software (Systat Software, Palo Alto, CA, USA). The data distribution was first evaluated for equal variance and normality (Shapiro–Wilk test). All statistical tests used (one-way ANOVA, one-way ANOVA on Ranks, and post-hoc Holm–Sidak) are reported in the main text and in the respective figure legends. The sample sizes (n) are reported in the figure legends. Significance was set at 0.05, and all tests were two-tailed. The results are reported as means ± SD.

## 3. Results

### 3.1. RI Induces Object-Memory Loss in Male but Not in Female C57bl/6 Mice

Vulnerability to RI may represent a key factor underlying gender-based differences in object recognition memory. To test this hypothesis, we used a variant of the NOR paradigm including an interference session (Figure 1A–C), adapted from the work of Liu et al. [24], and evaluated the effects of both proactive and retroactive interference on memory. In male mice, we observed a lower preference index (PI) in animals undergoing the RI paradigm compared to the standard NOR protocol (Std-NOR), whereas there were no differences between the Std-NOR and PrI groups (one-way ANOVA, F_(2;25)_ = 6.731; *p* = 0.005; PI: 65.7 ± 12.1% for Std-NOR, 52.7 ± 5.9% for RI, and 63.4 ± 5.9% for PI; Std-NOR vs. RI *p* = 0.007; Std-NOR vs. PI *p* = 0.604; Holm–Sidak post hoc test; Figure 1D). Instead, we found no differences in terms of PI among female mice undergoing the three different paradigms (one-way ANOVA, F_(2;25)_ = 0.590; *p* = 0.562; PI: 64.9 ± 8.2% for Std-NOR, 62.3 ± 13.0% for RI, and 67.7 ± 11.3% for PrI; Figure 1E). The exploration time during training, interference, and test did not significantly differ among the different experimental conditions (Table 1). Thus, our behavioral analyses pointed to a gender-specific vulnerability to RI-induced memory loss, with male mice being more susceptible than females.

### 3.2. ERα Is Crucial for Resistance to RI in Female Mice

**Figure 2:**
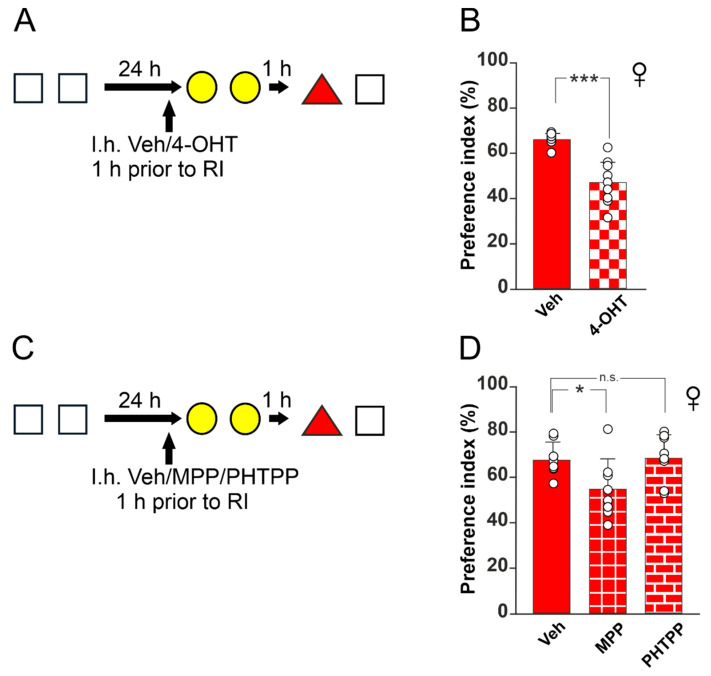
Estrogen receptors play a key role in resistance to retroactive interference. (**A**): Schematic representation of 4-hydroxytamoxifen (4-OHT) or vehicle (Veh) intrahippocampal (I.h.) injection during RI procedure; white squares represent object 1, yellow circles represent object 2, and red triangle represents object 3. (**B**): Histograms (means ± SD) showing preference indexes of animals undergoing RI after 4-OHT treatment. Note that 4-OHT injection induces memory loss. Asterisks indicate statistically significant differences between groups assessed by Kruskal–Wallis one-way analysis of variance on ranks (*p* < 0.001; Veh n = 8; 4-OHT n = 9). (**C**): Schematic representation of RI procedure with I.h. injection of Veh, MPP, or PHTPP, antagonists of the estrogen receptors α and β, respectively. (**D**): Histograms (means ± SD) showing percentages of PI in animals undergoing RI in the presence of Veh, MPP, or PHTPP. Asterisk indicates statistically significant difference between groups assessed by one-way ANOVA (F_(2;21)_ = 4.339; *p* = 0.026; Veh vs. PHTPP *p* = 0.906, Holm–Sidak post hoc test; Veh vs. MPP *p* = 0.042, Holm–Sidak post hoc test; n = 8 animals for each group). * *p* < 0.05; *** *p* < 0.001; n.s., not significant. All graphs and images were realized using CorelDraw21 (Corel Corporation, Ottawa, ON, Canada).

Once the gender-specific differences in the susceptibility to RI were established, we looked for molecular mechanisms underlying this effect, focusing on estrogen signaling. Specifically, we investigated whether a pharmacological downregulation of hippocampal estrogen receptor activity in female mice increased their vulnerability to RI. We focused on the dorsal hippocampus, considering that previous works documented the key role of this structure in murine models of object-induced interference [9]. Moreover, sex-dependent differences in hippocampal cognition have been ascribed to estrogen signaling [19]. First, we stereotaxically injected the antiestrogenic compound 4-hydroxytamoxyfen (4-OHT), an active metabolite of tamoxifen that selectively modulates estrogen receptors, into the dorsal hippocampus of female mice before the interference session (Figure 2A). The injection of 4-OHT enhanced the vulnerability of females to interference, as indicated by a significant reduction in PI upon 4-OHT injection compared to controls (47.4 ± 9.6% vs. 66.4 ± 2.8%, Kruskal–Wallis one-way analysis of variance on ranks, *p* < 0.001; Figure 2B). To determine whether this effect could be ascribed to estrogen receptors α (ERα) or β (ERβ), we also injected two other groups of female mice with either Erα- or Erβ-selective inhibitors, i.e., MPP and PHTPP, respectively (Figure 2C). The administration of PHTPP did not induce a significant interference-dependent change in memory performance in female mice, whereas MPP injection increased their vulnerability to the interference protocol and caused an impairment of object recognition memory compared to controls, thus suggesting a specific involvement of ERα (one-way ANOVA, F_(2;21)_ = 4.339; *p* = 0.026; PI: 68 ± 7.5% for vehicle-injected mice, 68.6 ± 10.4% for PHTPP-injected mice, 55 ± 13.2% for MPP-injected mice; vehicle vs. PHTPP *p* = 0.906; vehicle vs. MPP *p* = 0.042, Holm–Sidak post hoc test; Figure 2D). Exploration time during training, interference, and test did not differ among the experimental groups (Table 2). 

### 3.3. ERK1/2 Activation Plays a Critical Role in RI-Resistance of Female Mice

**Figure 3:**
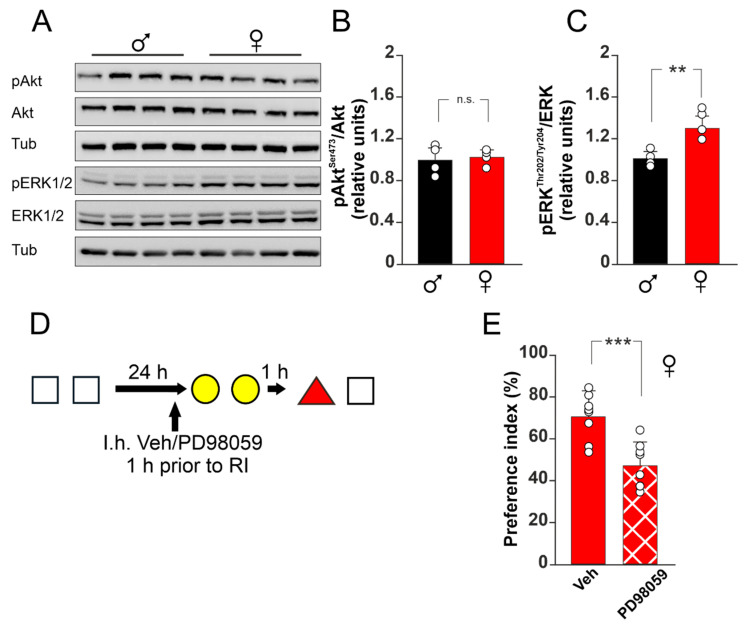
ERK1/2 inactivation leads to RI-induced memory loss in female mice. (**A**): Representative Western blots for Akt and ERK1/2 phosphorylation; white squares represent object 1, yellow circles represent object 2, and red triangle represents object 3. (**B**): Histograms (means ± SD) showing fold induction variation of Akt phosphorylation at S473. One-way ANOVA, F_(1;7)_ = 0.116; *p* = 0.745. n = 4 for both groups. (**C**): Histograms (means ± SD) showing fold induction variation of ERK1/2 phosphorylation at Thr202/Tyr204. One-way ANOVA, F_(1;7)_ = 16.885; *p* = 0.006; n = 4 for both groups. (**D**): Schematic representation of experimental protocols including vehicle (Veh) or PD98059 intrahippocampal (I.h.) injections. (**E**): Histograms (means ± SD) showing preference indexes of female mice undergoing the RI paradigm in the presence of vehicle or PD98059. Asterisks indicate statistically significant differences between groups assessed by one-way ANOVA (F_(1;14)_ = 18.368, *p* < 0.001; n = 8 for both groups). ** *p* < 0.01; *** *p* < 0.001; n.s., not significant. All graphs and images were realized using CorelDraw21 (Corel Corporation, Ottawa, Ontario, Canada).

Next, we asked about the downstream signaling molecules involved in the ERα-dependent resistance to RI. To address this issue, we assessed the phosphorylation levels of two kinases whose activation has been demonstrated in response to estradiol stimulation, i.e., protein kinase B (Akt) and ERK1/2. First, we analyzed the phosphorylation levels of these kinases in male and female mice after the interference paradigm. We found a statistically significant increase in the activating phosphorylation levels of ERK1/2 at Thr202/Tyr204 after the interference protocol in the hippocampus of female mice compared to male mice (one-way ANOVA, F_(1;7)_ = 16.885; *p* = 0.006; 30.2 ± 0.1% increase; Figure 3A,C). Conversely, no differences in the levels of the Akt-activating phosphorylation at S473 were observed between male and female mice (one-way ANOVA, F_(1;7)_ = 0.116; *p* = 0.745; Figure 3A,B). To confirm the critical role of ERK1/2 signaling in the interference-related vulnerability to memory loss, female mice were subjected to the interference paradigm after an intra-hippocampal injection of the ERK1/2 inhibitor PD98059 (Figure 3D). The administration of PD98059 significantly reduced the preference toward the novel object in interference paradigm in female mice compared to controls (one-way ANOVA, F_(1;14)_ = 18.368, *p* < 0.001; PI: 70.6 ± 11.0% for vehicle injected mice; 47.4 ± 10.8% for PD98059 injected mice; Figure 3E), suggesting that ERK1/2 activation plays a central role in mediating the resistance to RI-induced memory loss. This effect was independent on an inhibition of the memory retrieval process, as female mice injected with PD98059 (same timing and dosage used for RI) (Figure S1A) performed similarly to vehicle-injected mice in the Std-NOR paradigm (one-way ANOVA, F_(1;16)_ = 0.094; *p* = 0.763; Figure S1B). The exploration time during training, interference, and test did not differ among the conditions (Table 3).

## 4. Discussion

Our study investigated the gender differences in susceptibility to interference and the underlying molecular mechanisms. Our results show that sex hormones can influence memory performance in an object recognition task under RI conditions and that the estrogen signaling pathway plays a key role in this phenomenon. Specifically, we demonstrated that: (1) male mice are susceptible to object-related RI memory loss, whereas female mice are resistant; (2) the blockade of estrogen receptors by 4-OHT or the ERα antagonist MPP leads to RI-induced memory loss in female mice, confirming the key role of estrogens in this gender-specific effect; (3) RI-mediated memory loss relies on increased ERK1/2 phosphorylation, and, consequently, (4) ERK1/2 blockade leads to an increased susceptibility of female mice to the RI paradigm.

Memory-related sexual dimorphism can be ascribed to both differences in connections during development and the effects of estrogens on molecular determinants underlying information processing [14]. Sex-dependent differences in retroactive interference have been reported in human studies [29], although other studies reported conflicting results [30,31]. In these works, interference was administered right after the acquisition phase, and therefore during the consolidation phase, and subjects were tested shortly after. Previous studies reported that retroactive interference is maximally experienced when the interfering material is administered after the acquisition of the to-remember information or prior to the test phase [32,33,34]. We used a protocol in which interference was administered prior to the test phase, allowing us to evaluate the pharmacological modulation and molecular effects of interference separated from memory encoding and memory consolidation. Our behavioral results showed that in an object recognition task under RI or PrI conditions both genders were unaffected by the PrI paradigm, suggesting that the current protocol generates a memory that is probably not strong enough to induce PrI. However, the training memory was strong enough to be recalled at 24 h, as it can be observed in mice of both genders undergoing the Std-NOR paradigm, yet it is labile enough to be disrupted from newer learning. We did not observe any variations in the exploration time during the test phase of male and female mice, suggesting that the effect that we observed was independent of different exploratory behaviors between the sexes, as has been, instead, previously shown for object place paradigms [35]. It has been reported that females, during proestrus, which is characterized by high levels of circulating sex hormones such as 17β-estradiol (E2), show better object recognition memory when evaluated at a short time interval, even if other studies failed to reveal the same difference in both rats and mice [36,37,38,39,40,41,42]. However, the role of circulating E2 as the only determinant of gender differences in cognitive functions is debated [43] because both female and male rodents have been reported to locally produce estrogens inside the brain, especially in the hippocampus [44]. The inhibition of aromatase enzyme in the hippocampus halts LTP induction only in the female hippocampus [45], while it impairs working memory in male mice and rats and blocks object recognition memory and object place memory in gonadectomized male mice [46,47]. Furthermore, aromatase knockout male mice are reported to have impaired memory in social recognition tasks [48]. Therefore, these evidence collectively suggest that locally produced E2 is also crucial for cognitive functions in the male hippocampus and that estradiol represents not only a sex hormone but also an important synaptic player activating different receptors and signaling cascades in both sexes.

To unveil a role of estrogen signaling in the RI paradigm, we used 4-hydroxytamoxifen (4-OHT), an active metabolite of tamoxifen that selectively modulates estrogen receptors [49]. It has been reported that an intrahippocampal injection of tamoxifen interferes with memory retrieval, especially in paradigms based on spatial memory [50,51,52]. In our model, injections of 4-OHT into the dorsal hippocampus of female mice prior to the interference session induced memory loss, confirming the involvement of estrogen signaling in resistance to RI. The same effect was induced by an injection of the selective ERα antagonist MPP, whereas the selective ERβ antagonist PHTPP failed to affect memory. These findings suggest that ERα plays a key role in the hippocampal-dependent resistance to RI in male mice. Indeed, it has been reported that, in female mice, LTP induction in the hippocampus is dependent on the activity of Erα; in the presence of MPP but not PHTPP, the same stimulation protocol inducing LTP at the CA3-CA1 synapses in the hippocampus of male mice failed to do the same in female mice [53]. Moreover, in vivo studies showed that hippocampal ERα inhibition during the consolidation phase of an object displacement paradigm induces memory loss, whereas the hippocampal inhibition of ERβ during consolidation induces memory loss in both the object recognition and object displacement paradigms. These findings suggest that specific nuances of cognitive functions are modulated by different estrogen receptor subtypes, with a specific role of ERα for spatial memory consolidation [54]. Estrogen receptors are widely expressed in different brain regions subserving memory function, such as the prefrontal cortex (PFC), the amygdala, and the perirhinal cortex [21], where they may modulate cognition. For instance, the modulation of PFC activity has been demonstrated to be dependent on E2 concentration, as excessive or insufficient E2 levels impair its function [55]. The effects of E2 on cognition can also be dependent on the activity of a third receptor, the G-coupled ER, which has been shown to play a crucial role in modulating E2 effects, especially in the hippocampus of female mice [56]. Another sex-specific feature of estrogen signaling is related to GABAergic synapses into the hippocampus: in female mice, E2 can attenuate GABAergic responses by activating the glutamate metabotropic receptor 1 (mGluR1) molecular cascade, which has been reported to trigger ERK1/2 activation [57].

Downstream of the membrane-associated estrogen receptors, different kinases are recruited for synaptic function. Among these, we focused on ERKs because they have been shown to play a critical role in the hormonal regulation of memory function [58,59] and because the object memory improvement induced by exogenous estrogen administration triggers ERK1/2 activation in female mice but not in male mice [18]. Furthermore, since ERs lack any kinase activity, it has been reported that estrogen-dependent triggering downstream molecular cascades may depend on the strict association between membrane ERs and other receptors, such as mGluR1, insulin-like growth factor receptors, or brain-derived neurotrophic factor receptors [60,61,62,63]. It has been previously shown that the interaction between mGluR1 and ERα is functional only in the hippocampus of female mice and that mGluR1 activation can rapidly activate ERK1/2 signaling [62]. Indeed, when investigating the levels of activating phosphorylation of ERK1/2 in male and female mice undergoing the RI paradigm, we observed that females had significantly higher ERK1/2 Thr202/Tyr204 phosphorylation compared to males. Finally, we observed that the intrahippocampal blockade of ERK1/2 activation induced memory loss in females exposed to RI, suggesting a specific role for these kinases in regulating RI-vulnerability. ERK1/2 signaling is at the crossroad of many receptor signaling cascades, and its activity can be regulated in different manners based on the activating stimulus [64]. Indeed, ERK1/2 activity has been demonstrated to be necessary for most aspects of memory, from consolidation to reconsolidation to memory retrieval [65]. Thus, based on our findings, we can speculate that ERK1/2 activation supports the stabilization of the original memory trace [66] and promotes a pattern separation mechanism allowing the two memories to be stored independently.

Sex differences in cognition can be also observed in pathological settings such as Alzheimer’s disease (AD), for which women exhibit a higher risk [67]. Interestingly, different works have shown that patients suffering from AD are, in the early phases of the disease, far more susceptible to interference-mediated forgetting [68,69]. These results strengthen the idea that understanding circuital and molecular mechanisms underlying interference vulnerability could also give us a better comprehension of pathological mechanisms underlying disease progression. For example, the levels of interference vulnerability might also be used as a tool to help characterize AD progression and choose the therapeutic strategy. Indeed, few treatment options are presently available for AD, and none of them is differentiated for sex [70], while many compounds that are able to ameliorate memory, at least in preclinical models, work well in one sex and not in the other. For instance, vitamin A and its metabolite, retinoic acid, have been shown to modulate the effects of estrogen signaling by increasing aromatase and ERβ expression in rat hippocampal cultured slices [71] and by reducing tau hyperphosphorylation and amyloid toxic fragments in the hippocampus of male 3×Tg-AD mice, a widely used AD mouse model. This effect was further correlated with improved learning and memory abilities in the Y-maze [72]. However, the same phenomenon was not observed in age-matched 3×Tg-AD female mice, suggesting a clear sex-specific effect. Further studies are needed to understand whether in animal models of AD an estrogen-signaling-dependent vulnerability to interference may be differently regulated compared to wild-type animals and whether these differences can be translated to humans.

In conclusion, our study reveals a novel function of the hippocampal estrogenic molecular cascade involved in the gender-specific resistance to object-based RI, which, in female mice, relies on estrogen signaling and ERK1/2 activity within the dorsal hippocampus. Further studies will be necessary to elucidate the specific brain network involved in the interference mechanisms under both physiological and pathological conditions and to understand the differences in forgetting mechanisms in the context of gender differences.

## Figures and Tables

**Table 1:** Exploration time during training, interference, and test, relative to Figure 1. Std-NOR: standard novel object recognition; PrI: proactive interference; RI: petroactive interference.

Group	Training	Interference	Test
Males (Std-NOR; n = 9)	17.0 ± 5.8 s	not applicable	17.3 ± 6.1 s
Males (PI; n = 11)	18.2 ± 4.9.2 s	17.6 ± 4.6 s	16.6 ± 4.7 s
Males (RI; n = 8)	20.9 ± 5.9 s	20.7 ± 6.5 s	20.6 ± 6.6 s
Females (Std-NOR; n = 10)	15.8 ± 4.3 s	not applicable	16.2 ± 5.9 s
Females (PI; n = 10)	19.0 ± 5.6 s	17.6 ± 6.2	20.3 ± 3.1 s
Females (RI; n = 8)	18.6 ± 4.4 s	15.0 ± 6.5 s	15.7 ± 6.1 s

**Table 2:** Exploration time during training, interference, and test, relative to Figure 2. Veh: vehicle; 4-OHT: 4-hydroxytamoxifen; PHTPP: ER antagonist; MPP: ER antagonist.

Group	Training	Interference	Test
Veh (n = 8)	21.5 ± 13.2 s	not applicable	23.3 ± 10.0 s
4-OHT (n = 9)	23.5 ± 8.5 s	19.6 ± 7.1 s	21.6 ± 5.0 s
Veh (n = 8)	20.2 ± 5.8 s	24.7 ± 3.8 s	20.1 ± 4.4 s
MPP (n = 8)	22.3 ± 10.1 s	23.3 ± 11.6	21.3 ± 7.9 s
PHTPP (n = 8)	21.7 ± 2.4 s	22.4 ± 4.2	23.9 ± 5.2 s

**Table 3:** Exploration time during training, interference, and test, relative to Figure 3. Veh: vehicle; PD98059: MEK inhibitor.

Group	Training	Interference	Test
Veh (n = 8)	21.6 ± 5.0 s	not applicable	20.3 ± 6.1 s
PD98059 (n = 8)	20.3 ± 3.9 s	22.3 ± 8.9 s	23.4 ± 10.3 s

## Data Availability

The data are contained within the article and Supplementary Materials.

## Supplementary Materials

The following supporting information can be downloaded at: https://www.mdpi.com/article/10.3390/biomedicines10061387/s1, Figure S1: ERK1/2 inactivation does not influence memory retrieval in an Std-NOR paradigm.
