# Supplementary material for: Hippocampal Estrogen Signaling Mediates Sex Differences in Retroactive Interference
Source: Biomedicines. 2022 Jun 11;10(6):1387. doi: 10.3390/biomedicines10061387 (PMC9219958; doi:10.3390/biomedicines10061387)
Supplement: Supplementary file 1 [file biomedicines-10-01387-s001.zip › biomedicines-1761159-supplementary.pdf]

Supplementary Materials

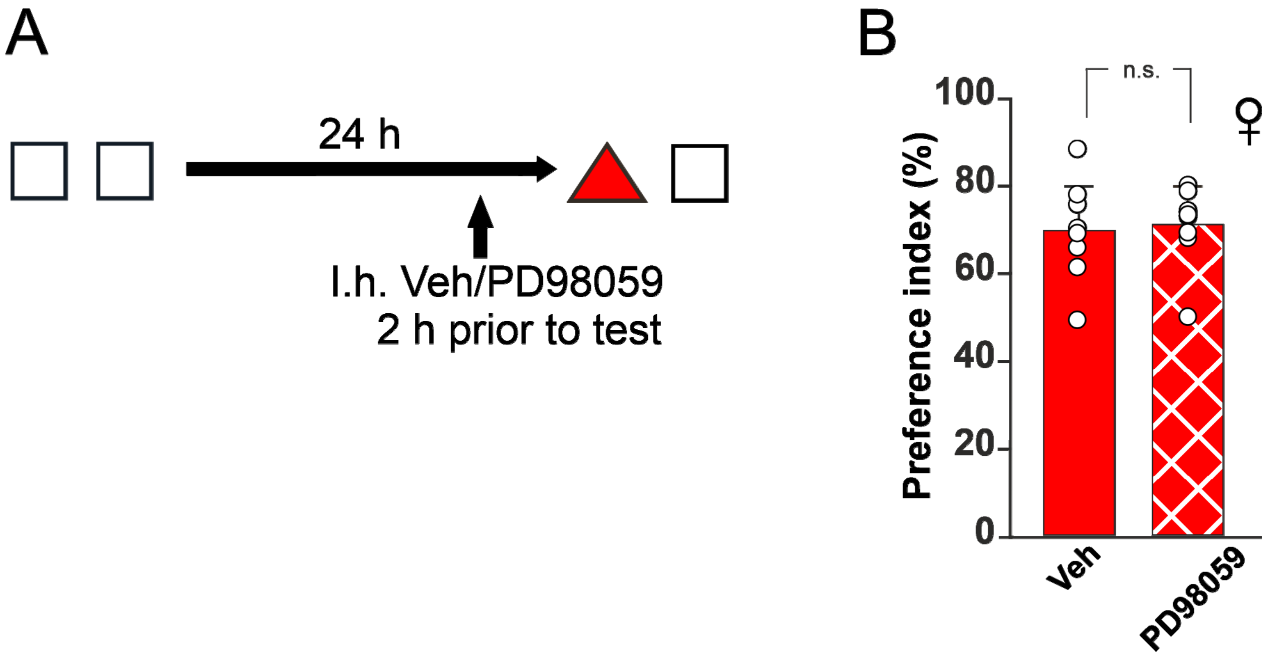

Supplementary Figure S1. ERK1/2 inactivation does not influence memory retrieval in a Std-NOR paradigm. A: Schematic representation of vehicle (Veh) or PD98059 intrahippocampal (I.h.) injection procedure; white squares represent object 1; red triangle represent object 2. B: Histograms (mean±SD) showing preference indexes of female mice undergoing the Std-NOR paradigm in the presence of vehicle or PD98059. One way ANOVA, $F_{(1;16)} = 0.094$; $p = 0.763$; Veh n = 8; PD98059 n = 9). n.s. not significant. All graphs and images were realized using CorelDraw21 (Corel Corporation, Ottawa, Ontario, Canada).
